# Acute Endobronchial Dissemination of a Tuberculoma Following EBUS‐GS‐Guided Transbronchial Biopsy: A Case Report

**DOI:** 10.1002/rcr2.70647

**Published:** 2026-06-18

**Authors:** Haruno Tsukagoshi, Yasuto Ueda, Tatsuya Nakada, Takashi Sumikawa, Yasuyuki Hasegawa, Mizuho Matsushita, Hiroki Chikumi, Akira Yamasaki

**Affiliations:** ^1^ Department of Respiratory Medicine Tottori Prefectural Central Hospital Tottori Japan; ^2^ Division of Medical Oncology and Molecular Respirology, Faculty of Medicine Tottori University Yonago Japan

**Keywords:** bronchoscopy, EBUS‐GS, endobronchial dissemination, tuberculoma, tuberculosis

## Abstract

Post‐bronchoscopy infections are usually bacterial, whereas procedure‐associated endobronchial dissemination of *Mycobacterium tuberculosis* is rarely reported. A 52‐year‐old woman with poorly controlled diabetes (HbA1c 13.8%) had a 19‐mm left upper‐lobe nodule radiologically suspicious for lung cancer. Because tissue confirmation was required and tuberculosis remained in the differential diagnosis (positive T‐SPOT.TB on admission), endobronchial ultrasonography with a guide sheath (EBUS‐GS)‐guided transbronchial biopsy was performed under airborne precautions. Bronchial lavage smear and loop‐mediated isothermal amplification were positive for *M. tuberculosis* , and histology showed extensive caseous necrosis with numerous acid‐fast bacilli, consistent with a tuberculoma. Two days later, she developed a 39°C fever with rapid cavitation of the primary lesion and new ipsilateral tree‐in‐bud opacities. No bacterial pathogens other than *M. tuberculosis* were identified despite empirical ceftriaxone. Standard antituberculosis therapy led to clinical and radiological improvement.

## Graphical abstract

Acute endobronchial dissemination highly suggestive of a procedure‐associated event occurred after EBUS‐GS‐guided biopsy of a pulmonary tuberculoma. Because tuberculoma may mimic lung cancer, clinicians should consider tuberculosis in solitary nodules and monitor carefully for dissemination after bronchoscopy when necrotic lesions are sampled.

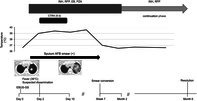

## 1 Introduction

Solitary pulmonary nodules may present a diagnostic dilemma because pulmonary tuberculoma can closely mimic primary lung cancer on imaging [1]. When tissue confirmation is required for radiologically indeterminate nodules, infectious aetiologies must remain in the differential diagnosis alongside malignancy. In Japan, tuberculoma accounts for less than 1% of resected solitary pulmonary nodules, though its clinical significance warrants careful consideration, particularly in patients with epidemiological risk factors [2]. Infectious complications after EBUS‐GS‐guided transbronchial biopsy are uncommon but recognised, with prior studies identifying lesion necrosis and larger lesion size as potential risk factors [3]. However, acute endobronchial dissemination of tuberculosis temporally associated with bronchoscopy has rarely been reported [4, 5]. We report a case of acute ipsilateral endobronchial dissemination following EBUS‐GS‐guided transbronchial biopsy of a pulmonary tuberculoma in a patient with severely uncontrolled diabetes.

## 2 Case Report

A 52‐year‐old woman was referred for evaluation and treatment of poorly controlled diabetes mellitus (random glucose 403 mg/dL; HbA1c 13.8% at referral). On admission, blood glucose was 286 mg/dL and HbA1c was 12.0% (Table 1). Three years earlier, her cohabiting mother‐in‐law had died from pulmonary tuberculosis. At that time, two interferon‐gamma release assays performed during contact screening were reportedly negative. Apart from diabetes‐related abnormalities, baseline laboratory findings were unremarkable. Chest radiography showed no apparent abnormality. Chest computed tomography (CT) revealed a 19‐mm irregular nodule in the left upper lobe, radiologically highly suspicious for primary lung cancer (Figure 1A,B). Although malignancy was the leading consideration, tuberculosis remained in the differential diagnosis because of the exposure history and a positive T‐SPOT.TB result on admission (Table 1). Because tissue confirmation was required, endobronchial ultrasonography with a guide sheath (EBUS‐GS)‐guided transbronchial biopsy was performed under airborne precautions. Bronchial lavage smear was positive for acid‐fast bacilli, and loop‐mediated isothermal amplification was positive for *Mycobacterium tuberculosis* . Histopathological examination demonstrated extensive caseous necrosis on haematoxylin and eosin staining and numerous acid‐fast bacilli on Ziehl‐Neelsen staining (Figure 2A,B), consistent with pulmonary tuberculoma. Sputum smear obtained the following day was also positive. The patient was hospitalised and started on standard four‐drug antituberculosis therapy with isoniazid, rifampicin, ethambutol and pyrazinamide. Although she had been asymptomatic before bronchoscopy, she developed a 39°C fever beginning 2 days after the procedure. The fever persisted intermittently for approximately 5 weeks after bronchoscopy. Because post‐bronchoscopy bacterial infection could not be excluded at fever onset, empirical ceftriaxone was administered for 5 days. However, no bacterial pathogens other than *M. tuberculosis* were identified from bronchial lavage fluid or sputum obtained the day after bronchoscopy, and ceftriaxone was discontinued. CT obtained approximately 10 days after bronchoscopy demonstrated cavitation of the original lesion and newly developed centrilobular nodules with a tree‐in‐bud appearance in the ipsilateral lower lobe (Figure 1C,D). Given the temporal association between the biopsy and the onset of fever 2 days later, the absence of pre‐existing dissemination on pre‐procedure CT, and the absence of bacterial pathogens despite empirical antibiotics, the radiological changes confirmed at approximately 10 days were considered highly suggestive of bronchoscopy‐associated endobronchial dissemination. After 2 months of intensive therapy followed by 4 months of continuation therapy with isoniazid and rifampicin, her symptoms resolved and radiographic abnormalities gradually improved. Sputum smears remained positive for 7 weeks after treatment initiation before subsequent conversion, supporting persistent active disease after dissemination. Complete radiological resolution was confirmed at month 6. Written informed consent for publication was obtained.

**TABLE 1:** Laboratory findings on admission.

Parameter	Value
Haematology	
WBC	6160 /μL
Neutrophils	73%
Monocytes	4.5%
Lymphocytes	20.5%
Eosinophils	1.3%
RBC	4.94 × 10^6^ /μL
Haemoglobin	13.9 g/dL
Platelets	32.8 × 10^4^ /μL
Biochemistry	
AST	12 U/L
ALT	17 U/L
LDH	178 U/L
BUN	17.4 mg/dL
Creatinine	0.50 mg/dL
Total Protein	6.7 g/dL
Albumin	3.4 g/dL
CRP	0.80 mg/dL
Sodium	135 mEq/L
Potassium	4.1 mEq/L
Calcium	9.2 mg/dL
Blood Glucose	286 mg/dL
HbA1c	12.0%
Infectious Disease Tests	
*β*‐D‐glucan	< 4.0 pg/mL
Cryptococcus Ag	Negative
Aspergillus Ag	0.2 (Negative)
T‐SPOT.TB	Positive
Tumour Markers	
CEA	2.63 ng/mL
SCC	0.9 ng/mL
ProGRP	26.1 pg/mL

Abbreviations: *β*‐D‐glucan, beta‐D‐glucan; Ag, antigen; ALT, alanine aminotransferase; AST, aspartate aminotransferase; BUN, blood urea nitrogen; CEA, carcinoembryonic antigen; CRP, C‐reactive protein; HbA1c, haemoglobin A1c; LDH, lactate dehydrogenase; ProGRP, pro‐gastrin‐releasing peptide; RBC, red blood cell count; SCC, squamous cell carcinoma antigen; T‐SPOT.TB, T‐SPOT.TB test; WBC, white blood cell count.

**FIGURE 1:**
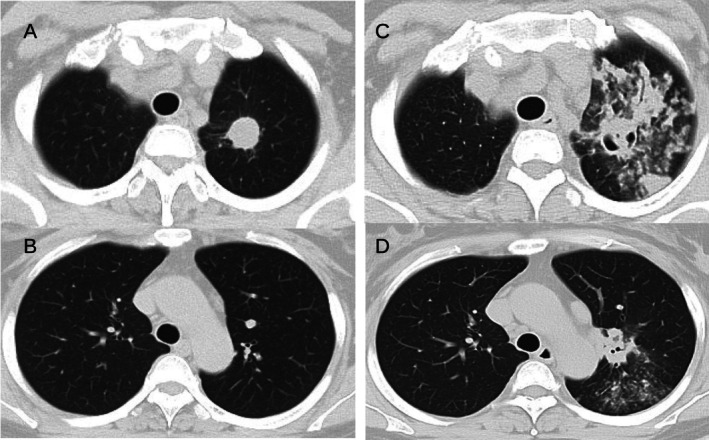
Chest computed tomography before and after EBUS‐GS‐guided transbronchial biopsy. (A, B) CT images obtained before bronchoscopy showed a solitary nodule confined to the left upper lobe without evidence of airway dissemination. (C, D) CT images obtained after bronchoscopy demonstrated cavitation of the original lesion and newly developed centrilobular nodules with a tree‐in‐bud appearance in the ipsilateral lower lobe, consistent with endobronchial dissemination. All images are displayed in lung window settings (window width 1500 HU, window level −600 HU). The cavitary wall and internal structure of the lesion were confirmed on mediastinal window images (not shown).

**FIGURE 2:**
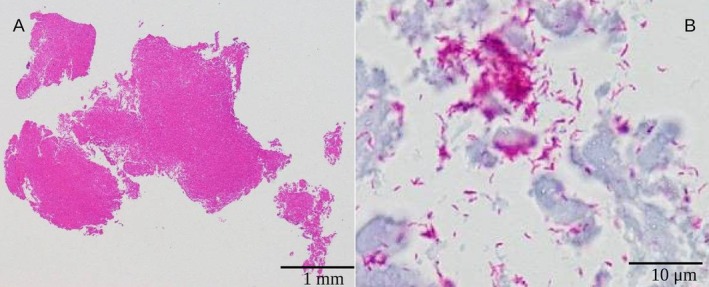
Histopathological findings of the transbronchial biopsy specimen. (A) Haematoxylin and eosin staining demonstrated extensive caseous necrosis with scant preserved lung architecture. (B) Ziehl‐Neelsen staining revealed numerous acid‐fast bacilli within the necrotic tissue.

## 3 Discussion

This case describes acute ipsilateral endobronchial dissemination that was highly likely associated with EBUS‐GS‐guided biopsy of a pulmonary tuberculoma. The short interval from bronchoscopy to the onset of high fever, rapid cavitation of the primary lesion and newly developed ipsilateral tree‐in‐bud opacities strongly support a procedure‐associated event. Importantly, the patient had been asymptomatic before bronchoscopy, and pre‐procedure CT showed no evidence of airway dissemination, making spontaneous acute progression less likely. In addition, no bacterial pathogens other than *M. tuberculosis* were identified despite empirical antibiotic therapy, making secondary bacterial pneumonia less likely.

Previous reports of post‐bronchoscopy worsening of tuberculosis are limited. Cui et al. described acute dissemination after bronchoalveolar lavage [4], and Tsang et al. reported rapid enlargement of a tuberculous cavity after bronchoscopy [5]. In contrast, our case involved EBUS‐GS‐guided biopsy of a peripheral tuberculoma, suggesting a risk related to direct mechanical manipulation of a necrotic, bacillary‐rich lesion through a guide sheath. To our knowledge, this is the first reported case of endobronchial dissemination following EBUS‐GS‐guided biopsy of a tuberculoma. Hiraishi et al. reported that respiratory complications occurred in 0.9% of patients undergoing EBUS‐GS‐guided biopsy for peripheral pulmonary lesions, and all three affected patients had cavitary lesions on CT, suggesting that mechanical disruption of necrotic or cavitary tissue during bronchoscopy may facilitate intrabronchial spread of infectious material [6].

The procedural indication was appropriate: tissue confirmation was required for a nodule with malignant features, while tuberculosis remained a meaningful differential given the exposure history and positive T‐SPOT.TB, prompting EBUS‐GS under airborne precautions.

Host and lesion factors likely contributed to the rapid progression. The biopsy specimen showed extensive necrosis with numerous acid‐fast bacilli, suggesting a high bacillary burden. Severely uncontrolled diabetes may impair macrophage‐ and T‐cell‐mediated immunity and destabilise tuberculous granulomas, potentially facilitating rapid progression when necrotic material is mechanically disrupted [7]. This case also raises the practical question of whether better glycaemic control before an invasive diagnostic procedure might reduce the risk of progression or dissemination in similar high‐risk patients. From a procedural perspective, repeated instrument passes through the guide sheath and lavage may have mobilised caseous material and distributed it to adjacent or distal segments of the ipsilateral lung.

Practical lessons from this case are as follows: (i) tuberculosis should remain in the differential diagnosis of solitary peripheral nodules, even when malignancy is suspected; (ii) when EBUS‐GS is performed for necrotic lesions in which tuberculosis is possible, clinicians should recognise the potential risk of dissemination and monitor closely after bronchoscopy; and (iii) persistent new‐onset fever following bronchoscopy, especially when unresponsive to empirical antibiotic therapy, combined with new tree‐in‐bud opacities on CT, should prompt evaluation for endobronchial dissemination of tuberculosis in addition to bacterial infection.

## Author Contributions

Haruno Tsukagoshi and Yasuto Ueda drafted the manuscript. Tatsuya Nakada, Takashi Sumikawa, Yasuyuki Hasegawa, Mizuho Matsushita, Hiroki Chikumi and Akira Yamasaki contributed to clinical management and data acquisition. All authors read and approved the final manuscript.

## Funding

The authors have nothing to report.

## Ethics Statement

Ethics approval was not required for this case report; written informed consent for publication was obtained.

## Consent

The authors declare that written informed consent was obtained for the publication of this manuscript and accompanying images using the consent form provided by the Journal.

## Conflicts of Interest

The authors declare no conflicts of interest.

## Data Availability

The data that support the findings of this study are available from the corresponding author upon reasonable request.

## References

[1] J. Cao , Y. Gu , X. C. Wu , et al., “EBUS‐GS With the GeneXpert MTB/RIF Assay for Diagnosis of *Mycobacterium tuberculosis* Infection of Isolated Pulmonary Nodules,” European Journal of Medical Research 28 (2023): 370. 37742037 10.1186/s40001-023-01331-z PMC10517488

[2] N. Hosaka , M. Kameko , H. Nishimura , and S. Hosaka , “Prevalence of Tuberculosis in Small Pulmonary Nodules Obtained by Video‐Assisted Thoracoscopic Surgery,” Respiratory Medicine 100, no. 2 (2006): 238–243. 15964180 10.1016/j.rmed.2005.05.004

[3] T. Souma , T. Minezawa , H. Yatsuya , et al., “Risk Factors of Infectious Complications After Endobronchial Ultrasound‐Guided Transbronchial Biopsy,” Chest 158 (2020): 797–807. 32145245 10.1016/j.chest.2020.02.025

[4] Y. Cui , B. Wang , and C. Yang , “Imaging of Acute Dissemination of Tuberculosis Caused by Alveolar Lavage in Endobronchial Tuberculosis With Caseous Necrosis,” Respirology Case Reports 12 (2024): e70059. 39479222 10.1002/rcr2.70059 PMC11522607

[5] K. W. Tsang , B. Jones , and I. P. Mungall , “A Case of Rapidly Expanding Tuberculous Lung Cavity After Bronchoscopy,” Respiratory Medicine 91 (1997): 377–379. 9282242 10.1016/s0954-6111(97)90066-4

[6] Y. Hiraishi , T. Izumo , S. Sasada , et al., “Factors Affecting Bacterial Culture Positivity in Specimens From Bronchoscopy in Patients With Suspected Lung Cancer,” Respiratory Investigation 56, no. 6 (2018): 457–463. 30392535 10.1016/j.resinv.2018.07.006

[7] B. I. Restrepo , “Diabetes and Tuberculosis,” Microbiology Spectrum 4, no. 6 (2016): TBTB2‐0023‐2016. 10.1128/microbiolspec.TNMI7-0023-2016 PMC5240796 28084206

